# Integrated photothermal microcarriers for precise exosome‐secreted microRNA profiling in breast cancer diagnosis

**DOI:** 10.1002/qub2.58

**Published:** 2024-06-21

**Authors:** Yunjie Shi, Yun Cheng, Peiyu Chen, Lexiang Zhang, Fangfu Ye

**Affiliations:** ^1^ School of Pharmaceutical Sciences Wenzhou Medical University Wenzhou China; ^2^ Oujiang Laboratory (Zhejiang Lab for Regenerative Medicine, Vision and Brain Health) Wenzhou Institute University of Chinese Academy of Sciences Wenzhou China; ^3^ Department of Pediatrics The Second Affiliated Hospital and Yuying Children’s Hospital of Wenzhou Medical University Wenzhou China; ^4^ Joint Centre of Translational Medicine the First Affiliated Hospital of Wenzhou Medical University Wenzhou China; ^5^ Beijing National Laboratory for Condensed Matter Physics Institute of Physics Chinese Academy of Sciences Beijing China

**Keywords:** breast cancer, digital PCR, exosomes, MicroRNA, photothermal

## Abstract

Breast cancer constitutes a significant global health burden, while conventional diagnosis approaches may lack precision and can be discomforting for patients. Exosomes have emerged as promising biomarkers for breast cancer due to their participation in diverse pathological processes, and a convenient analysis platform is believed to greatly promote its application. In this study, we propose a novel digital PCR approach utilizing near‐infrared (NIR) photo‐responsive thermosensitive microcarriers integrated with black phosphorus for quantifying microRNA (miRNA) biomarkers within exosomes. Petal‐like biomimetic nanomaterials were firstly assembled for non‐specific exosome capture based on the affinity effect of avidin and biotin. Photothermal‐responsive microcarriers, fabricated using gelatin‐based substrates blended with photothermal nanocomposite, exhibited NIR‐induced heating and reversible phase transition properties. We optimized synthesis parameters on thermal response and established a programmable and controllable NIR light source module. The results indicated a significant elevation in the levels of biomarkers miRNA‐1246 and miRNA‐122, with fold increases ranging from 6.2 to 23.6 and 5.9 to 13.0, respectively, in breast cancer cell lines MCF‐7 and MDA‐MB‐231 compared to healthy control cells HUVEC. This study offers broad prospects for utilizing exosomes to resolve predictive biomarkers.

## 1 INTRODUCTION

Breast cancer, a prevalent malignancy, constitutes a significant portion of global cancer cases and ranks among the primary causes of cancer‐related mortality in women [1, 2]. Timely surgical intervention facilitates early detection; however, conventional diagnostic approaches, such as biopsies and imaging modalities, may lack precision, and invasive procedures can be discomforting, thereby complicating disease management [3]. Exosomes are nano‐sized extracellular vesicles (EVs) secreted by mammalian cells, which play a significant role in intercellular communication by ferrying various macromolecular cargos, including microRNA (miRNA), lncRNA, proteins, and lipids [4, 5]. Single tumor cells release over 10^4^ exosomes daily, which are intricately linked to diverse physiological and pathological processes, indicating their potential as non‐invasive biomarkers for breast cancer [6]. Notably, these exosomes significantly influence the tumor microenvironment and expedite breast cancer metastasis [7]. For instance, transmission of exosome‐associated miRNA‐19a to osteoclasts led to breast cancer metastasis to bone, regarded as an incurable complication [8]. Exosomes containing miRNA‐1246 from breast cancer cells transferred resistance to chemotherapeutic agents like cisplatin, docetaxel, and paclitaxel, thereby promoting drug resistance [9]. Additionally, the low levels of let‐7 exosome release, prompted by the pluripotent factor, induced an immunosuppressive response, thereby regulating neutrophil count and N2 transformation [10]. Thermal activity sensor hence employed tracking eight specific proteins on exosomes to monitor metastatic breast cancer progression [1]. Despite promising discoveries, clinical application of these technologies is hindered by specialized exosome manipulation infrastructures and intricate characterization techniques [11, 12, 13].

We propose a novel method utilizing near‐infrared (NIR) photo‐responsive thermosensitive microcarriers integrated with black phosphorus (BP) to quantify miRNA biomarkers within exosomes. Digital PCR (dPCR) is an advanced molecular technique used for precise quantification of nucleic acids [14, 15]. Unlike traditional PCR formats, dPCR partitions one sample into numerous independent sub‐reactions, which achieves exceptional precision and absolute quantification with resistance to diverse PCR inhibitors, making it suitable for challenging clinical sample types [11]. With enhanced diagnostic capabilities, it has been popularized for biodiversity assessment, rare target detection, and diverse applications [16, 17, 18]. Recent advancements have seen dPCR revolutionizing diagnostic possibilities such as in combination with microfluidic chips for particles and agents capturing and pretreatment, immuno‐dPCR for detecting barcoded sequences, integrating with photothermal materials for light‐triggered thermocycling pattern, etc [19, 20]. Quadruple color‐coded dPCR technology allowed simultaneous analysis of PUM1, PGR, ESR1, and ERBB2 oncogenes by separating droplet chambers to avoid amplification interference, successfully classifying multiple pathological subtypes such as triple negative and luminal A [21]. The integration of microwell array technology with nucleic acid enrichment and single‐molecule digital detection enabled precise quantification of RNA molecules [22]. Our proposed approach aims to develop a non‐invasive, highly sensitive, and user‐friendly early breast cancer diagnostic by incorporating BP composite nanomaterials into phase‐changeable microcarriers' gel network and employing NIR radiation for intelligent dPCR. Its potential in improving diagnostic accuracy and patient outcomes warrants further exploration.

We introduce a new breast cancer diagnostic strategy based on exosomal miRNA profiling, feasible for laboratory benchtop implementation. Initially, we established a nonspecific exosome isolation protocol using biomimetic petal‐shaped magnetic nanoparticles (NPs). Subsequently, the isolated exosomes, thermosensitive gel bases, and photothermal nanocomposite premix were emulsified to form uniform microcarriers. A calibrated NIR light program facilitated thermal cycling, followed by fluorescence signal counting using a flow cytometer instrument. NPs offer surface modification versatility and have been incorporated into diverse biomedical nanoplatforms, along with detecting ctDNA signatures, assisting cancer theranostics, co‐delivery drugs and active agents, forming engineered biomaterials, and the like [23, 24, 25, 26, 27, 28]. By leveraging biotin‐avidin affinity, the decorated NPs indiscriminately captured exosomes, preserving the integrity of the exosome RNA library. The synthesis of capture NPs involved the deposition of a silica layer on the surface of Fe_3_O_4_ cores, followed by catalyzing the outer layer into magnesium silicate in an alkaline environment. This morphology is advantageous for trapping nanoscale membranous vesicles. After constructing responsive microcarriers, we analyzed synthesis parameters' response patterns for efficient photothermal heating up rate and reversible sol‐gel phase transition. A homemade programmable NIR light source enabled automated PCR thermal cycling process. This experimental platform effectively distinguished two types of breast cancer cells from healthy control cells by quantifying the relative expression levels of target miRNA molecules. These findings unveil new avenues for predicting biomarkers and quantifying cell‐to‐cell signaling using exosomes.

## 2 RESULTS AND DISCUSSION

### 2.1 Biomimetic nanomaterials for non‐specific capture of exosomes

**FIGURE 1:**
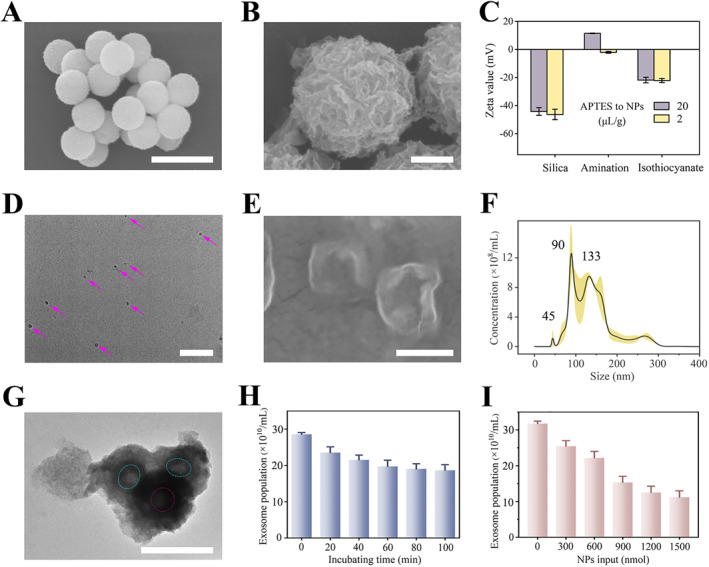
Biomimetic petal‐shaped magnetic nanomaterials non‐specifically captured the exosomes. (A) SEM image of synthesized Fe_3_O_4_ NPs. (B) SEM image of the petal‐like shape of the synthesized Fe_3_O_4_@MgSiO_3_ NPs. (C) Zeta potential characterization after modification of amination, and thiocyanate conjugation. Morphological characteristics of exosome model samples in (D–E) SEM images at different magnifications and (F) particle size distribution. (G) TEM image of exosomes captured by the material. Capture efficiency as a function of (H) blending time and (I) capture particle usage. Scale bar, 500 nm in (A and G), 300 nm in (B and E), and 1 μm in (D). NPs, nanoparticles. SEM, scanning electron microscopy. TEM, transmission electron microscopy.

To obtain a comprehensive RNA library from exosomes, we designed efficient affinity connections between biotin and avidin on the outer surface of the capture materials to achieve non‐specific enrichment of exosomes in the reaction solution. Specifically, we first synthesized monodisperse Fe_3_O_4_ NPs with a particle size of 300 nm using the hydrothermal method (Figure 1A). Then, an approximately 50 nm layer of silica was deposited on their surface using an improved Stöber method to provide flexible surface chemical treatment and excellent biocompatibility. Subsequently, in an ammonia solution, a hydrothermal reaction with metal ions resulted in the gradual deposition and stacking of silicates on the outer surface, forming a petal‐like biomimetic structure (Figure 1B), thereby greatly increasing the contact surface area and capture capacity. Once the desired morphology of the capture particles was achieved, affinity capture was accomplished by sequentially hydroxylation, amination modification, and thiocyanate functional group connection on the surface. The Zeta potential of the material was measured after each chemical modification step (Figure 1C). If the amination activation step was insufficient, indicated by the Zeta potential remaining negative, it would result in inadequate loading of thiocyanate and final avidin. Consequently, even after modification, the Zeta potential would still exhibit negative values.

Once assembling the morphology and chemical modification of the particles, they were mixed with exosome model samples to quantitatively assess the capture efficiency. We first adopted ultracentrifugation technology to extract exosomes from cell culture supernatants and used them as model samples to evaluate the capture ability of nanomaterials. The obtained exosomes exhibited a discoid morphology with a median concave feature and vesicle sizes ranging from around 50 to 200 nm, with 1 × 10^12^ vesicles per group collected (Figure 1D–F). NPs ranging from 300 to 1500 nmoL in quantity were incubated with exosomes (Figure 1G), and 3 μL of supernatant was collected at 20‐min intervals to measure exosome abundance using a NTA instrument. The difference between adjacent samples allowed us to deduce the change in capture efficiency over time and particle dosage. Generally, increasing the particle dosage and incubation time within the range of 300–1200 nmoL and 20–60 min, respectively, significantly enhanced efficiency (Figure 1H–I). However, further increases in these parameters yielded limited additional benefits. Therefore, we selected the optimized conditions of 1200 nmoL dosage and 60 min of incubation time to conduct separation experiments from two breast cancer cell lines MCF‐7 and MDA‐MB‐231, as well as the healthy control group HUVEC. Subsequently, internal nucleic acid libraries were analyzed using dPCR.

**FIGURE 2:**
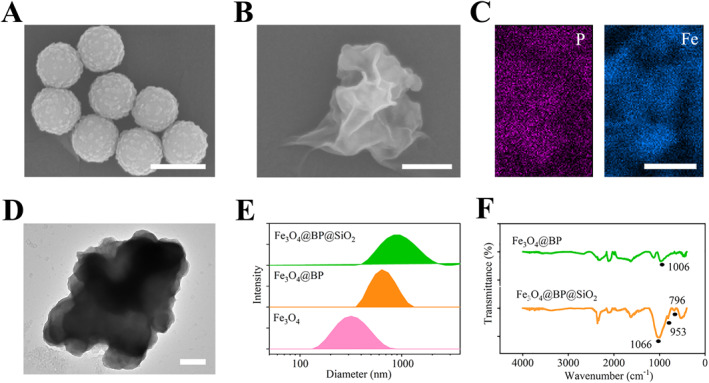
Controllable development of photothermal responsive composite nanomaterials. SEM images of (A) Fe_3_O_4_ NPs and (B) Fe_3_O_4_@BP composite. (C) Elemental analysis indicating the distribution of P and Fe elements in Fe_3_O_4_@BP composite. (D) TEM image of Fe_3_O_4_@BP@SiO_2_ composite. (E) Size distribution and (F) FTIR spectrum characterization of the material and intermediates. Scale bar, 500 nm in (A and D), 150 nm in (B), and 200 nm in (C). NPs, nanoparticles; SEM, scanning electron microscopy; TEM, transmission electron microscopy.

To achieve a light‐driven temperature control mode for the samples, we continued to design and assemble a photo‐responsive composite nanomaterial. Specifically, we chose Fe_3_O_4_ NPs as the dispersion carrier (Figure 2A). Then a layer of polydopamine was loaded as an adhesive to Sandwich a sheet‐like monolayer of BP on the outer surface (Figure 2B,C), enhancing the surface area for light absorption and also preventing the two‐dimensional material from aggregating into clusters due to hydrogen bonding and van der Waals forces during heating. Electron microscopy and nanoparticle size analysis revealed that thorough stirring during the synthesis process controlled the internal encapsulated spheres population and achieved uniform sizes. Finally, a simplified Stöber reaction was employed to deposit a thin layer of silica on the outer surface (Figure 2D–E), rendering the material compatible with nucleic acid molecules and enzymes and preventing adsorption of nucleic acid molecules due to *π*‐*π* stacking effects, which could hinder PCR. The good light transmittance of the silica coating also allowed the BP within the composite material to sense light irradiation and generate heat, with a significantly higher heating effect compared to the Fe_3_O_4_@SiO_2_ group without the BP layer. Comparison of fourier transform infrared (FTIR) spectra between intermediates in the stepwise assembly process demonstrated the distinct peak at 1006 cm^−1^, attributed to carboxyl groups in BP, was clearly visible in the Fe_3_O_4_@BP material (Figure 2F). The peak disappeared upon complete coverage by the silica layer, indicating an effective combination of components as designed.

### 2.2 Controlled preparation and performance study of the microcarriers

**FIGURE 3:**
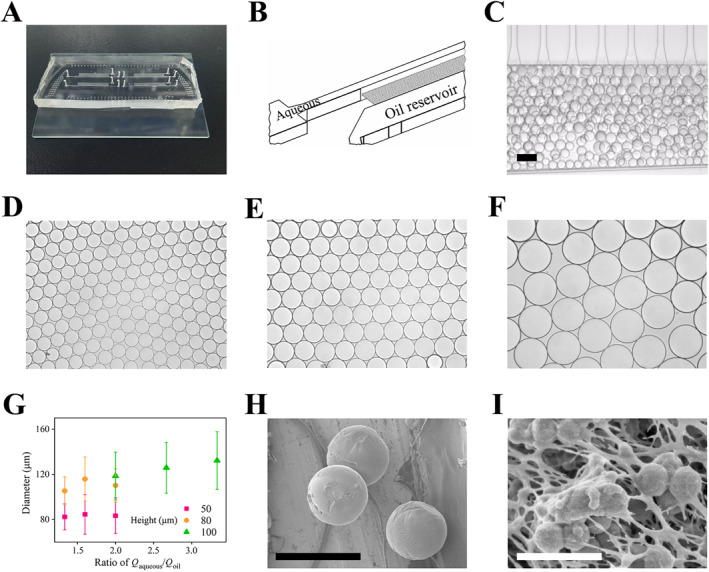
Illustration of the controlled development of responsive microcarriers. (A) A photo of the millipede‐structured microfluidic chip, (B) a schematic diagram detailing its characteristic structure, and (C) the experimental process of producing emulsified microcarriers. (D–F) Monodispersed microcarrier groups with tuned sizes, and (G) their relationship with biphasic flow rates and channel step depth. (H–I) SEM images of the microcarriers, with partial magnification showing the composite nanomaterials loaded within the gel network. Scale bar, 100 μm in (C), 300 μm in (H), and 1 μm in (I). SEM, scanning electron microscopy.

We utilized a 4% (w/v) gelatin‐based substrate mixed with photothermal materials and PCR reaction reagents to prepare high‐throughput gel microcarrier arrays on a microfluidic chip featuring parallel step emulsion structures (Figure 3A–C). Specifically, a photolithography method was employed to assemble the step structures by dual exposure of two aligned patterns with different depths. Within each unit structure, the reaction solution dispersed into parallel microchannels, with the fluid front cascading down the steps through fan‐shaped nozzle exit structures to form droplets in the oil reservoir. A significant enhancement in fabrication throughput, up to several mL/min, was achieved with 100 parallel units. By adjusting key parameters such as the flow rates of the aqueous and oil phase, as well as the depth of the step, we controlled the microspheres with diameters ranging from 80 μm to 140 μm and deviations around 20% range (Figure 3D–G), which were characteristic dimensions commonly used in the dPCR technology. Droplet size was significantly controlled by the depth of the steps, while it was relatively insensitive to the flow rate of the oil phase. After the microcarriers solidifying into gel, dehydration and drying were conducted to ensure scanning electron microscopy (SEM) characterization. Specifically, we soaked the collected microcarriers in gradient ethanol solvents from 50% to 100% for several stages, which allowed for gradual dehydration. Finally, they were dried in pure ethanol solvent, CO_2_ protective atmosphere and 10°C environment for 4 hours to eliminate crystal water and maintain the spherical shape intact. This characterization revealed the complete sphericity and monodispersity of the sizes, along with the uniform dispersion of the composite nanomaterial within them (Figure 3H–I).

**FIGURE 4:**
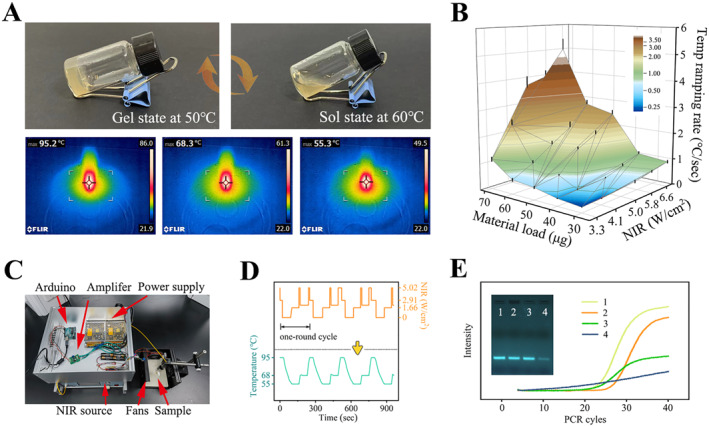
Investigation on the intelligent responsiveness and biocompatibility of gelatin microcarriers assembled with Fe_3_O_4_@BP@SiO_2_ composite. (A) NIR illumination facilitated sol‐gel reversible phase transition. The photothermal effect enabled it to heat up temperature at 95, 55, and 68°C as the PCR denaturation, annealing, and extension stages. (B) The heating up rate of 50 μL microcarrier emulsions under NIR was influenced by the concentration of Fe_3_O_4_@BP@SiO_2_ addition and the NIR intensity. (C) A photo of the self‐constructed NIR light source module controlled via Arduino chip programming. (D) The implemented NIR program and associated samples undergoing four cycles of typical PCR thermal cycling. (E) Gelatin concentration on PCR amplification efficiency was evaluated through qPCR curves and electrophoresis gel bands of the resulting products. Sample numbers 1–4 represent gelatin concentrations of 0%, 4%, 8%, and 16% respectively. NIR, near‐infrared.

The gelatin‐based substrate blended with composite materials exhibited characteristics of NIR light‐induced heating and simultaneously maintained the inherent phenomenon of gel‐sol reversible phase transition of gelatin at approximately 55°C (Figure 4A). Then an in‐depth quantitative investigation was conducted on the photothermal response characteristics of microcarriers concerning NIR intensity and the loading capacity of nanocomposite materials (Figure 4B). It is worth noting that the heating rate of the photothermal effect during the PCR annealing, which could easily reach 1.5–2°C/s when rising from 55°C to 95°C, with negligible variation between repeated tests. Compared with traditional Peltier and other heat conduction and temperature changing technologies involving reaction solutions, tubes and metal heating blocks, this light‐responsive method features by deploying heat sources inside the reagents, endowed with the advantages of noncontact and low energy consumption. Cooling speed of the prepared fan set was twice that of natural cooling, achieving an effect close to 1°C/s.

To achieve repetitive illumination and automatic switching between different cycling stages, we developed a programming control module based on Arduino circuit board to assist the NIR light source (Figure 4C). The NIR light source was sequentially connected to the input/output (I/O) ports, linear voltage amplifier, and Arduino open‐source chip, with both the I/O serial line and the voltage amplifier powered by two voltage switches. Virtual voltage commands generated by the Arduino program were transmitted to the voltage amplifier, where output to the NIR laser source and fans via the I/O port interface, thereby achieving precise control over light intensity and radiation time. When placing 50 μL microcarrier emulsions within the NIR light spot, the heating processes from 55°C to 95°C and 68–95°C only took around 30 s and 8 s, respectively. In this manner, the three temperature stages corresponding to the denaturation, annealing, and extension steps in the PCR thermal cycle were decomposed into specific NIR light‐mediated heating, temperature maintenance, and cooling modes in series. Microcarriers exhibited extremely high sensitivity to NIR activation/deactivation and adjustment conditions. By gradually calibrating temperature stages, we successfully assembled a commonly used PCR thermal cycling process, demonstrating stable temperature responses over four cycles (Figure 4D). These research findings demonstrated the high repeatability and controllability of dPCR microcarriers in NIR responsiveness, thus laying a solid foundation for temperature‐sensitive enzyme‐catalyzed biological reactions mediated by NIR.

### 2.3 Profiling the expression of exosomal miRNA in dysregulated states

**FIGURE 5:**
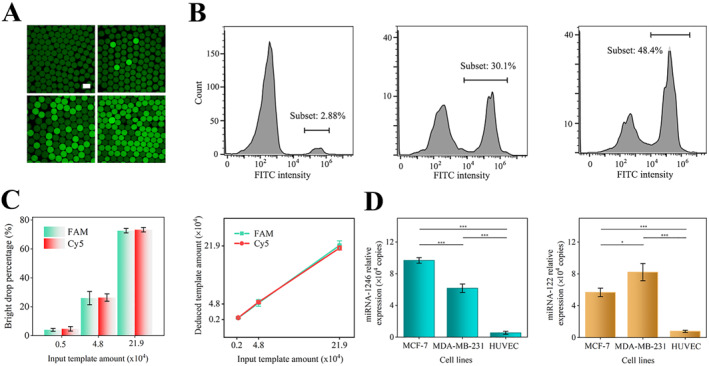
Detection performance of target molecules via photothermal dPCR. (A) Confocal laser microscope images illustrate the bright drop percentages with target molecule concentrations ranging from 0 to 1.5 copies per droplet. (B) Statistical and distribution analysis on the fluorescence intensity of microcarriers with different miRNA concentrations using flow cytometry. (C) The proportion of detected fluorescent droplets across samples with varying miRNA concentrations. The miRNA abundance was subsequently deduced from the experimentally derived fluorescent droplet percentages. (D) Relative expression levels of miRNA‐1246 and miRNA‐122 biomarkers within exosomes secreted by MCF‐7, MDA‐MB‐231, and HUVEC cells. Scale bar, 100 μm in (A).

Given the evidence linking miRNA dysregulation to early screening and progression of breast cancer, we plan to employ photothermal dPCR technology to investigate the expression of key target genes. Among the selected miRNA targets, exosomal miRNA‐1246 was widely recognized for downregulating Cyclin G2 expression, leading to the breast cancer progression [29, 30]. Additionally, exosomal miRNA‐122 was validated to reprogram glucose metabolism and promote metastasis in breast cancer [9]. To achieve PCR amplification and fluorescence labeling of miRNA molecules as short as 23 nt, we first hybridized them with circular single‐stranded probes under isothermal conditions, extending them to a length of 66 nt, then utilizing respective primers and TaqMan probes with FAM and Cy5 fluorophores to label different targets. Further observation of fluorescent droplets confirmed the successful amplification process, where the 14 nt circular structure and the 6 nt pairing region between the miRNA molecule could support the connection of a 30 nt fragment. After the dPCR process, fluorescent microcarriers harbored the target miRNA and suggested successful amplification, whereas non‐fluorescent microcarriers indicated the absence of the target molecule (Figure 5A). In this study, we chose to fabricate microcarriers with a diameter of 83 μm, which was corresponding to equivalent to emulsifying approximately 167,100 populations from 50 μL of reaction solution. This size ensured smooth passage through flow cytometry instruments with a nozzle diameter of 150 μm. Flow cytometry has the capability to rapidly detect proportions of fluorescent agents with statistical significance. By statistically analyzing the fluorescence values of individual microcarriers (Figure 5B), two distinct fluorescence intensity peaks were identified: one around 300 and another around 2 × 10^5^. These peaks corresponded to dark and fluorescent microcarrier groups, respectively, and a threshold can be easily set to divide them.

We gradually diluted the input templates and measured the proportion of detected fluorescent microcarriers, based on which a standard curve reflecting specificity and linearity was constructed (Figure 5C). The experimentally deduced initial template amount was derived from statistical regression analysis using the Poisson distribution. Statistically, if the proportion of fluorescent microcarriers is less than 10%, then positive microcarriers mostly contain only one target molecule, while those containing two or more target molecules are rare. When the proportion of fluorescent microcarriers approaches 10%, multiple target molecules may be randomly distributed into a single positive droplet. Specifically, using Poisson statistics, we can infer that 73% of fluorescent microcarriers in 167,100 population correspond to the numbers of microcarriers containing 1, 2, 3, or more than 3 target molecules as 59,060, 38,680, 16,900, and 7,370, respectively. When template concentration reaches high levels deviating from the linear relationship with gradient dilution, it is generally recommended to pre‐dilute before quantification within a stable linear range. The detection limit of this droplet digital technology is typically determined by the detection level of blank samples without templates. Here, we adopt the threshold of 0.3% of the bright droplet percentage, which was the mean of three blank sample tests plus three times the standard deviation.

Two breast cancer cell lines MCF‐7 and MDA‐MB‐231, as well as one healthy cell HUVEC, were cultured in equivalent cell numbers, and exosomes from the supernatant were collected in the identical experimental condition. Subsequently, the copy numbers of miRNA targets were quantified using photothermal dPCR. Notably, miR‐1246 and miR‐122 were detected as 6.2–23.6 and 5.9–13.0 folds higher in the breast cancer exosomes, respectively, compared to the control ones (Figure 5D). The minute *p*‐values and standard deviations among triplicated tests revealed the significance of these differences, indicating the ability of photothermal dPCR to detect 5‐fold minor changes. This study elucidates the significant potential of exosomal miRNA in non‐invasive diagnosis of breast cancer.

## 3 CONCLUSION

In recent years, due to the combined progress and integration of materials science, nucleic acid enrichment technologies, and advanced biochemical techniques, numerous efficient diagnostic methods have emerged in the disease diagnosis field. Particularly, in the realm of intelligent responsive materials and biomimetic structural design, a range of innovative solutions have been provided. With the increasing demand for accuracy in breast cancer cell diagnosis, there is an urgent need for a platform capable of efficiently handling exosome biomarkers, which will aid in better guiding, optimizing, and evaluating the efficacy of therapeutic interventions.

We have successfully developed gelatin microcarriers doped with BP composite and biomimetic petal‐shaped exosome‐affinitive magnetic beads. Based on these technologies, we constructed a thermosensitive microcarrier dPCR method dependent on NIR light response for precise quantification of expression levels of exosomal miRNAs. Our constructed microcarriers pose sol‐gel transition characteristics, which not only addressed the issue of easy coalescence of traditional water droplets but also provided new possibilities for photothermal‐driven thermal cycling. In the sol state, the material met the diffusion and amplification needs and effectively dispersed the functional nanomaterials. Additionally, after gel formation, it could efficiently store and retain nucleic acid products and fluorescent signals internally, facilitating the efficient reading of fluorescent microspheres by flow cytometer instruments, and even possible for selecting positive microcarriers for further analysis of the internal amplicons. These intelligent microcarriers still maintained the advantages of droplet digitalization, exhibiting a low detection limit and the ability to efficiently distinguish minor changes of around 6‐fold. Furthermore, we designed a portable and programmable NIR light source module aimed at achieving accessible, cost‐effective, and automated photothermal thermal cycling processes. These features provide a new direction for accurately delineating the expression profiles of oncogenic miRNAs in exosomes.

## 4 EXPERIMENTAL SECTION

### 4.1 Materials

Products purchased from SYLGARD, 3M Novec, RAN Biotechnologies, Scientific Commodities, and Sigma companies included polydimethylsiloxane (PDMS 184), HFE 7500 oil, fluorinated surfactants, connecting tubes, gelatin, dopamine hydrochloride, mineral oil, Tween 20, Tris·buffer, and dodecyl gallate and acetate. PCR solutions, bovine serum albumin, PCR, loop primers, and TaqMan probes were gained from ABclonal, Biotopped, Shanghai Sangon Biotech, and Shanghai Jerry. Monolayer BP dispersion, tetraethoxysilane, pyridine, ammonia solution, magnesium chloride, ammonium chloride, avidin, biotinylated DSPE‐PEG, 3‐aminopropyltriethoxysilane, and phenyl isothiocyanate were purchased from Xilong Scientific, Nanjing Xianfeng Nanomaterials, Life Technologies, Freshwater Science and Technology, Beijing OKA, Energy Chemical, and Shanghai Maokang Biotechnology. Anhydrous ethanol and hydrochloric acid were purchased from China Pharmaceutical Chemical Company. Seeedstudio, SLQXF, and Studing were gained for Arduino circuit boards, voltage amplifiers, and fan components. Deionized water products were obtained from the Millipore Milli‐Q Plus system in this study.

### 4.2 Characterization

We employed transmission electron microscopy (TEM) (FEI) and SEM (SU8010) techniques for morphological observation and in‐depth analysis of elemental mapping. Prior to SEM imaging, samples were spin‐coated with a platinum layer to enhance their conductivity, with the accelerating voltages set at 5–10 kV (SEM) and 100–200 kV (TEM) during operation. The working principle of the Nanophox Zetasizer, a nanoparticle size analyzer, involves determining the size range by averaging 10 samples of the specimen. By utilizing nanoparticle tracking analysis (Malvern NS300) and continuous frame image processing technology with an injection pump, we accurately represented relevant information such as the size of extracellular vesicles. Infrared spectroscopy measurements were conducted using an FTIR spectrometer (Tensor II). Observation during the microsphere fabrication process on microfluidic chips was carried out using an Olympus microscope (BX53) and high‐speed camera (Optronis). Techniques such as PCR, qPCR, and gel electrophoresis were performed using the Bio‐Rad equipment system. We utilized flow cytometry to collect signals from FAM and Cy5 fluorescence channels of microspheres and conducted statistical analysis.

### 4.3 Cells and exosomes preparation

MCF‐7 cells were cultured in MEM medium supplemented with 100 units/mL of penicillin, 100 μg/mL of streptomycin, and 10% exosome‐depleted fetal bovine serum (HyClone). The exosome removal process involved pre‐treatment with 120,000 g ultracentrifugation for 12 h. MDA‐MB‐231 and HUVEC cells were cultured in a similar environment, with the base medium replaced with L15 and high glucose DMEM respectively. All cells were maintained in a humidified incubator at 37°C and 5% CO_2_. To remove dead cells, cell debris, and large molecules, the culture supernatant underwent sequential centrifugation at 300 g for 10 min, 2000 g for 10 min, and 100,000 g for 50 min, followed by ultracentrifugation at 120,000 g for 90 min after filtration through a 0.2 μm filter.

### 4.4 Synthesis method of petal‐shaped nanoparticles for exosome capture

Fe_3_O_4_@SiO_2_ NPs were synthesized using a hydrothermal method and optimized Stöber technique. Initially, 0.3 mmoL of MgCl_2_·6H_2_O and 3.5 mmol of NH_4_Cl were mixed in 10 mL of water, followed by the addition of 0.5 mL of concentrated ammonia solution (28%) and the dispersion of Fe_3_O_4_@SiO_2_ NPs. After a 14‐h hydrothermal reaction at 140°C, cleaning was performed using ethanol. Subsequently, the particles were immersed in a 10 mL solution of 0.1% sodium hydroxide for 30 min to activate surface hydroxyl groups. Next, 20 mg of NPs were mixed with 0.5 mL of aminopropyltriethoxysilane in 3 mL of concentrated ammonia solution, followed by dispersion in 5 mL of anhydrous ethanol and continuous mixing at 300 rpm for 3 h. Then, 10 mg of NPs and 0.05 g of phenyl isothiocyanate were added to 36 mL of dimethylformamide containing 4 mL of pyridine, followed by 2 h of stirring to graft isothiocyanate on the particle surface. Zeta potential was measured at each step of surface modification, with the transition between positive and negative values considered as a core signal reflecting the efficiency of each processing step.

### 4.5 Functionalized NPs enable the nonspecific isolation of exosomes

In an ethanol solution, 0.002 g of DSPE‐PEG‐labeled biotin, at a concentration of 1 mmol/L, was added. Initially, 100 μL of the exosome model sample was diluted five times with Diluent C buffer, then mixed with 0.5 mL of Diluent C buffer containing 10 nmoL of DSPE‐PEG‐biotin. Subsequently, incubation at 4°C for 1 h allowed NeutrAvidin to be immobilized on the capture particles modified with isothiocyanate. To achieve closure, 1% BSA was added. Finally, exosomes labeled with biotin were mixed with freshly NeutrAvidin‐modified capture particles, and 3 μL samples were extracted at specific incubation time points for nanoparticle tracking analysis to quantify the capture efficiency.

### 4.6 Photothermal dPCR

Complete exosomal libraries containing miRNAs were extracted from captured particle samples using a commercial kit and purified to a 60 μL aqueous environment. Initially, DNA molecules in the reaction mixture were degraded, followed by the addition of 4 μL of reverse transcription premix and 2 μM of stem‐loop primers to obtain a 50 μL reaction mixture. The entire reverse transcription process was incubated at 50°C for 12 min followed by 85°C for 5 min. Subsequently, 10 μL of cDNA was mixed with 0.3 μM of forward and reverse primers, opto‐thermal nanocomposite materials, and TaqMan probe, forming a 50 μL PCR reaction mixture. By emulsifying the premix in HFE7500 oil phase containing surfactants using a microfluidic chip with centipede structures, we obtained microsphere carriers of the desired diameter. The emulsion samples underwent PCR thermal cycling operations guided by an Arduino program, specifically, 31–33 cycles of 95°C for 30 s, 55°C for 60 s, and 68°C for 60 s. Finally, fluorescent signals of microspheres were characterized and quantified using flow cytometry and confocal microscopy, with each experiment repeated three times to obtain statistically analyzed results.

### 4.7 Synthesis of Fe_3_O_4_@BP@SiO_2_

The synthesis of Fe_3_O_4_@BP@SiO_2_ involved exploiting the adhesive properties of polydopamine to initially coat a thin layer of BP onto the surface of nanoparticle clusters. Specifically, a mixture comprising 2 mL of Fe_3_O_4_ NPs dispersion (0.3 g/mL), 6.5 mL of Tris·HCl buffer solution (pH 8.5), and 1.5 mL of dopamine hydrochloride (13.3 mg/mL) was prepared, resulting in a total reaction volume of 10 mL. Subsequently, the reaction mixture was stirred for 6 h in an open glass container, leading to a significant color change to black. After removing the supernatant by centrifugation, the NPs were uniformly dispersed in a 10 mL BP solution. The dispersion underwent continuous stirring for an additional 6 h through alternating cycles of vertexing and ultrasonication, ultimately yielding the Fe_3_O_4_@BP composite material. Finally, a thin layer of silica was deposited on the surface using the Stöber method, thus forming the specific Fe_3_O_4_@BP@SiO_2_ structure.

### 4.8 Statistical analysis

All quantified characteristics were defined as the mean with standard deviation (*n* = 3). *p*‐values were calculated using one‐way analysis of variance and Tukey’s multiple comparison test. Statistical significance was defined as indicated by * for *p* ≤ 0.05, ** for *p* ≤ 0.01, and *** for *p* ≤ 0.001.

## AUTHOR CONTRIBUTIONS

**Yunjie Shi**: Data curation; investigation; writing – original draft. **Yun Cheng**: Data curation; investigation; resources; writing – original draft. **Peiyu Chen**: Data curation; investigation. **Lexiang Zhang**: Conceptualization; supervision; writing – original draft. **Fangfu Ye**: Conceptualization; funding acquisition; supervision; writing – review & editing.

## CONFLICT OF INTEREST STATEMENT

The authors declare no competing financial interests.

## ETHICS STATEMENT

This study was approved by the Ethical Committee of the Wenzhou Institute, University of Chinese Academy of Sciences.

## Data Availability

The data that support the findings of this study are available from the corresponding author upon reasonable request.

## References

[1] Li Y , Zhang S , Liu C , Deng J , Tian F , Feng Q , et al. Thermophoretic glycan profiling of extracellular vesicles for triple‐negative breast cancer management. Nat Commun. 2024;15(1):2292. 38480740 10.1038/s41467-024-46557-5 PMC10937950

[2] Nolan E , Lindeman GJ , Visvader JE . Deciphering breast cancer: from biology to the clinic. Cell. 2023;186(8):1708–1728. 36931265 10.1016/j.cell.2023.01.040

[3] Will M , Liang J , Metcalfe C , Chandarlapaty S . Therapeutic resistance to anti‐oestrogen therapy in breast cancer. Nat Rev Cancer. 2023;23(10):673–685. 37500767 10.1038/s41568-023-00604-3 PMC10529099

[4] Wu B , Liu D.‐A , Guan L , Myint PK , Chin L , Dang H , et al. Stiff matrix induces exosome secretion to promote tumour growth. Nat Cell Biol. 2023;25(3):415–424. 36797475 10.1038/s41556-023-01092-1 PMC10351222

[5] Iannotta D , Kijas AW , Rowan AE , Wolfram J . Entry and exit of extracellular vesicles to and from the blood circulation. Nat Nanotechnol. 2024;19(1):13–20. 38110531 10.1038/s41565-023-01522-z PMC10872389

[6] Bao H , Tian Y , Wang H , Ye T , Wang S , Zhao J , et al. Exosome‐loaded degradable polymeric microcapsules for the treatment of vitreoretinal diseases. Nat Biomed Eng. 2023:1–17. 37872369 10.1038/s41551-023-01112-3

[7] Shin H , Choi BH , Shim O , Kim J , Park Y , Cho SK , et al. Single test‐based diagnosis of multiple cancer types using Exosome‐SERS‐AI for early stage cancers. Nat Commun. 2023;14(1):1644. 36964142 10.1038/s41467-023-37403-1 PMC10039041

[8] Wu K , Feng J , Lyu F , Xing F , Sharma S , Liu Y , et al. Exosomal miR‐19a and IBSP cooperate to induce osteolytic bone metastasis of estrogen receptor‐positive breast cancer. Nat Commun. 2021;12(1):5196. 34465793 10.1038/s41467-021-25473-y PMC8408156

[9] Li XJ , Ren ZJ , Tang JH , Yu Q . Exosomal MicroRNA MiR‐1246 promotes cell proliferation, invasion and drug resistance by targeting CCNG2 in breast cancer. Cell Physiol Biochem. 2018;44(5):1741–1748. 10.1159/000485780 29216623

[10] Qi M , Xia Y , Wu Y , Zhang Z , Wang X , Lu L , et al. Lin28B‐high breast cancer cells promote immune suppression in the lung pre‐metastatic niche via exosomes and support cancer progression. Nat Commun. 2022;13(1):897. 35173168 10.1038/s41467-022-28438-x PMC8850492

[11] Zhang K , Cheng K . Stem cell‐derived exosome versus stem cell therapy. Nat Rev Bioeng. 2023;1(9):608–609. 10.1038/s44222-023-00064-2 PMC10092910 37359776

[12] Zhang X , Zhang H , Gu J , Zhang J , Shi H , Qian H , et al. Engineered extracellular vesicles for cancer therapy. Adv Mater. 2021;33(14):2005709. 10.1002/adma.202005709 33644908

[13] Qin Y , Ge G , Yang P , Wang L , Qiao Y , Pan G , et al. An update on adipose‐derived stem cells for regenerative medicine: where challenge meets opportunity. Adv Sci. 2023;10(20):2207334. 10.1002/advs.202207334 PMC10369252 37162248

[14] Nan L , Zhang H , Weitz DA , Shum HC . Development and future of droplet microfluidics. Lab Chip. 2024;24(5):1135–1153. 38165829 10.1039/d3lc00729d

[15] Zhang L , Parvin R , Fan Q , Ye F . Emerging digital PCR technology in precision medicine. Biosens Bioelectron. 2022;211:114344. 35598553 10.1016/j.bios.2022.114344

[16] Guo W , Tao Y , Liu W , Song C , Zhou J , Jiang H , et al. A visual portable microfluidic experimental device with multiple electric field regulation functions. Lab Chip. 2022;22(8):1556–1564. 35352749 10.1039/d2lc00152g

[17] He H , Yang C , Wang F , Wei Z , Shen J , Chen D , et al. Mechanically strong globular‐protein‐based fibers obtained using a microfluidic spinning technique. Angew Chem Int Ed. 2020;59(11):4344–4348. 10.1002/anie.201915262 31873970

[18] Guo L , Cao M , Chen X , Zhang L , Zhang X , Zou L , et al. One‐step fabrication of microfluidic W/O/W droplets as fat‐reduced high internal phase emulsions: microstructure, stability and 3D printing performance. Food Hydrocolloids. 2024;150:109742.

[19] Liu C , Lin H , Guo J , Yang C , Chen J , Pan W , et al. Profiling of single‐vesicle surface proteins via droplet digital immuno‐PCR for multi‐subpopulation extracellular vesicles counting towards cancer diagnostics. Chem Eng J. 2023;471:144364.

[20] Hou Y , Chen S , Zheng Y , Zheng X , Lin JM . Droplet‐based digital PCR (ddPCR) and its applications. TrAC, Trends Anal Chem. 2023;158:116897.

[21] Chen W , Zheng J , Wu C , Liu S , Chen Y , Liu X , et al. Breast cancer subtype classification using 4‐Plex droplet digital PCR. Clin Chem. 2019;65(8):1051–1059. 31010819 10.1373/clinchem.2019.302315

[22] Wang D , Wang X , Ye F , Zou J , Qu J , Jiang X . An Integrated amplification‐free digital CRISPR/Cas‐assisted assay for single molecule detection of RNA. ACS Nano. 2023;17(8):7250–7256. 37052221 10.1021/acsnano.2c10143

[23] Yi K , Wang X , Filippov SK , Zhang H . Emerging ctDNA detection strategies in clinical cancer theranostics. Smart Med. 2023;2(4):e20230031. 39188296 10.1002/SMMD.20230031 PMC11235813

[24] Zhou W , Ma X , Wang J , Xu X , Koivisto O , Feng J , et al. Co‐delivery CPT and PTX prodrug with a photo/thermo‐responsive nanoplatform for triple‐negative breast cancer therapy. Smart Med. 2022;1:e20220036. 39188747 10.1002/SMMD.20220036 PMC11235718

[25] Fan L , Wang L , Wang X , Zhang H . Exosomes‐based particles as inhalable COVID‐19 vaccines. Biomed Technol. 2023;4:24–27.

[26] Furtado M , Chen L , Chen Z , Chen A , Cui W . Development of fish collagen in tissue regeneration and drug delivery. Eng Regen. 2022;3:217–231.

[27] Rajora AK , Ahire ED , Rajora M , Singh S , Bhattacharya J , Zhang H . Emergence and impact of theranostic‐nanoformulation of triple therapeutics for combination cancer therapy. Smart Med. 2024;3(1):e20230035. 39188518 10.1002/SMMD.20230035 PMC11235932

[28] Mustafa RA , Parkkila P , Rosenholm JM , Zhang H , Viitala T . Monitoring silica core@ shell nanoparticle‐bacterial film interactions using the multi‐parametric surface plasmon resonance technique. Smart Med. 2023;2(3):e20230012. 39188349 10.1002/SMMD.20230012 PMC11236032

[29] Fong MY , Zhou W , Liu L , Alontaga AY , Chandra M , Ashby J , et al. Breast‐cancer‐secreted miR‐122 reprograms glucose metabolism in premetastatic niche to promote metastasis. Nat Cell Biol. 2015;17(2):183–194. 25621950 10.1038/ncb3094 PMC4380143

[30] Yan W , Cao M , Ruan X , Jiang L , Lee S , Lemanek A , et al. Cancer‐cell‐secreted miR‐122 suppresses O‐GlcNAcylation to promote skeletal muscle proteolysis. Nat Cell Biol. 2022;24(5):793–804. 35469018 10.1038/s41556-022-00893-0 PMC9107513

